# Antineoplastic effects of clove buds (*Syzygium aromaticum* L.) in the model of breast carcinoma

**DOI:** 10.1111/jcmm.13197

**Published:** 2017-05-19

**Authors:** Peter Kubatka, Sona Uramova, Martin Kello, Karol Kajo, Peter Kruzliak, Jan Mojzis, Desanka Vybohova, Marian Adamkov, Karina Jasek, Zora Lasabova, Pavol Zubor, Silvia Fialova, Svetlana Dokupilova, Peter Solar, Martin Pec, Katarina Adamicova, Jan Danko, Mariusz Adamek, Dietrich Busselberg

**Affiliations:** ^1^ Department of Medical Biology Jessenius Faculty of Medicine Comenius University in Bratislava Martin Slovakia; ^2^ Division of Oncology Biomedical Center Martin Jessenius Faculty of Medicine Comenius University in Bratislava Martin Slovakia; ^3^ Department of Obstetrics and Gynecology Jessenius Faculty of Medicine Comenius University in Bratislava Martin Slovakia; ^4^ Department of Pharmacology Faculty of Medicine P. J. Šafárik University Košice Slovakia; ^5^ Department of Pathology Slovak Medical University and St. Elisabeth Oncology Institute Bratislava Slovakia; ^6^ Department of Chemical Drugs Faculty of Pharmacy University of Veterinary and Pharmaceutical Sciences Brno Czech Republic; ^7^ Department of Anatomy Jessenius Faculty of Medicine Comenius University in Bratislava Martin Slovakia; ^8^ Department of Histology and Embryology Jessenius Faculty of Medicine Comenius University in Bratislava Martin Slovakia; ^9^ Department of Pharmacognosy and Botany Faculty of Pharmacy Comenius University in Bratislava Bratislava Slovakia; ^10^ Department of Pharmaceutical Analysis and Nuclear Pharmacy Faculty of Pharmacy Toxicological and Antidoping Center Comenius University in Bratislava Bratislava Slovakia; ^11^ Institute of Biology and Ecology Faculty of Science Laboratory of Cell Biology P. J. Safarik University Kosice Slovakia; ^12^ Department of Pathological Anatomy Jessenius Faculty of Medicine Comenius University in Bratislava Martin Slovakia; ^13^ Department of Thoracic Surgery Faculty of Medicine and Dentistry Medical University of Silesia Katowice Poland; ^14^ Qatar Foundation‐Education City Weill Cornell Medicine in Qatar Doha Qatar

**Keywords:** mammary carcinogenesis, rat, cloves, cancer stem cells, epigenetics, angiogenesis, apoptosis, cell proliferation, MCF‐7 cells

## Abstract

It is supposed that plant functional foods, rich in phytochemicals, may potentially have preventive effects in carcinogenesis. In this study, the anticancer effects of cloves in the *in vivo* and *in vitro* mammary carcinoma model were assessed. Dried flower buds of cloves (CLOs) were used at two concentrations of 0.1% and 1% through diet during 13 weeks after the application of chemocarcinogen. After autopsy, histopathological and immunohistochemical analyses of rat mammary carcinomas were performed. Moreover, *in vitro* evaluation using MCF‐7 cells was carried out. Dietary administered CLO caused the dose‐dependent decrease in tumour frequency by 47.5% and 58.5% when compared to control. Analysis of carcinoma cells in animals showed bcl‐2, Ki67, VEGFA, CD24 and CD44 expression decrease and Bax, caspase‐3 and ALDH1 expression increase after high‐dose CLO administration. MDA levels were substantially decreased in rat carcinomas in both CLO groups. The evaluation of histone modifications revealed increase in lysine trimethylations and acetylations (H4K20me3, H4K16ac) in carcinomas after CLO administration. TIMP3 promoter methylation levels of CpG3, CpG4, CpG5 islands were altered in treated cancer cells. An increase in total RASSF1A promoter methylation (three CpG sites) in CLO 1 group was found. *In vitro* studies showed antiproliferative and pro‐apoptotic effects of CLO extract in MCF‐7 cells (analyses of cytotoxicity, Brdu, cell cycle, annexin V/PI, caspase‐7, Bcl‐2 and mitochondrial membrane potential). This study showed a significant anticancer effect of clove buds in the mammary carcinoma model *in vivo* and *in vitro*.

## Introduction

It is well documented that regular consumption of phytochemicals from whole foods (functional foods) is linked with a risk reduction of the diseases of civilization, including cancer 1. Plethora of studies demonstrated that phenolics, carotenoids and other plant chemicals display anticancer and several other biological activities, for example antioxidant, anti‐inflammatory or immunomodulatory 2. Many mechanisms of action have been shown to account for the anticancer actions of dietary phytochemicals, for example proliferation, apoptosis, angiogenesis, cancer stem cells (CSCs), without eliciting undesirable side effects in cells. Disruptions of epigenetic characteristics including hypermethylation of tumour suppressor gene promoters, aberrant chemical modification of histones or global DNA hypomethylation have been considered hallmarks of carcinogenesis 3. Recent preclinical research showed significant effects of dietary phytochemicals on epigenetic characteristics with important role in the transformation of normal to neoplastic cells 4.

Several recent epidemiological studies revealed that long‐term consumption of plant‐derived functional foods is linked with a decreased risk of breast carcinoma 5, 6, 7. CLOs (*Syzygium aromaticum L*.) are spices which consist of a mixture of phytochemicals—phenolic acids, flavonol glucosides, tannins and phenolic volatile oils (eugenol, acetyl eugenol) with the highest antioxidant activity among plant functional foods 8, 9. Besides acting as a strong antioxidant, clove buds also show other beneficial effects, for example antiproliferative, anti‐inflammatory, antibacterial and antiseptic, which make this food a potentially suitable natural substance for cancer chemoprevention 8. The positive activity of clove's extract in terms of anticancer effects in breast carcinoma has been documented in only a few *in vitro* studies. Different extracts of CLOs (*S. aromaticum*) demonstrated significant cytotoxic activity against several cancer cell lines, including MDA‐MB‐231 and MCF‐7 breast carcinoma cell lines 10. In another study, different concentrations of water and ethanol extract and essential oil of CLOs (*S. aromaticum*) were evaluated for antitumour potential *in vitro* using MTT assay and a brine shrimp lethality test. Authors concluded that CLOs showed excellent cytotoxicity towards MCF‐7 cells 8.

Anticancer effects of CLOs have not been assessed in animal mammary carcinoma model so far. The goal of this experiment was the evaluation of chemopreventive effects of dietary administered CLOs in N‐methyl‐N‐nitrosourea s(NMU)‐induced rat mammary gland cancerogenesis. The effects of CLOs on the mechanism of action (apoptosis, proliferation, angiogenesis, CSCs and epigenetics) in mammary carcinoma cells of the rats were evaluated. To confirm the anticancer effects of CLOs observed *in vivo* and gain more reproducible data for human population, a parallel *in vitro* study using human adenocarcinoma cells (MCF‐7) was realized.

## Material and methods

The experiment was approved by the Ethical Commission of the Jessenius Faculty of Medicine of Comenius University (Protocol No. EK1125/2012) and by the State Veterinary and Food Administration of the Slovak Republic (accreditation no. Ro‐1759/11‐221).

### Animals and induction of mammary carcinogenesis, design of experiment

Sprague‐Dawley female rats (Charles River Laboratories, Sulzfeld, Germany) aged 32**‐**36 days were acclimatized to standard vivarium conditions with temperature 23 ± 2°C, relative humidity 40–60%, artificial regimen (L/D 12 : 12 hrs). During the experiment, the animals were fed the Ssniff^®^ R‐Z low‐phytoestrogen V1354‐0 diet (Soest, Germany) and drinking water *ad libitum*. Mammary carcinogenesis was induced by N‐nitroso‐N‐methylurea (NMU, Sigma‐Aldrich, Deisenhofen, Germany) administered intraperitoneally (single dose of 50 mg/kg bodyweight on average on the 42nd postnatal day). This model mimics high‐risk premenopausal women 11.

Chemoprevention with CLOs (*S. aromaticum* L.) (Calendula, Nová Ľubovňa, Slovak Republic; country of origin—Madagascar/Comoros) began 1 week before carcinogen administration and lasted until 13 weeks after NMU administration. CLOs (ground flower buds) were administered in the diet (milled and processed by ‘cold pelleting procedure’) at two concentrations of 1 g/kg (0.1%) and 10 g/kg (1%). Animals (*n* = 25 per group) were randomly assigned into three experimental groups: (*i*) control group without chemoprevention; (*ii*) chemoprevention with CLOs at a concentration of 0.1% (CLO 0.1); (*iii*) chemoprevention with CLOs at a concentration of 1% (CLO 1). The animals were weighed and palpated weekly to register the presence, number, location and size of each palpable tumour. Food intake during 24 hrs was monitored in the 7th and 13th weeks of the experiment (the average daily dose of CLOs per rat was 19.24 mg in the CLO 0.1 and 200.5 mg in the CLO 1). In the last (13th) week of the experiment, the animals were quickly decapitated, the blood from each animal was collected, mammary tumours were excised, and the tumour size was recorded.

### Histopathological and immunohistochemical analysis of rat tumours

A tissue sample of each mammary tumour was routinely formalin‐fixed and paraffin‐embedded. The tumours were classified according to the criteria for the classification of rat mammary tumours 12. The additional parameter—grade of invasive carcinomas—was used. Tumour samples were divided into low‐grade (LG) and high‐grade (HG) carcinomas. The criteria for categorization (solidization, cell atypia, mitotic activity index and necrosis) were chosen according to the standard diagnostic method of classification. HG carcinomas were considered to be tumours with ≥2 positive criteria; LG carcinomas were tumours with ≤1 positive criterion 13, 14. Serum metabolic parameters (total cholesterol, very low‐density lipoprotein cholesterol, low‐density lipoprotein cholesterol, high‐density lipoprotein cholesterol, triacylglycerols, glucose) were evaluated using an Olympus AU640 (Olympus Optical, Tokyo, Japan) automatic biochemical analyser.

The most relevant part of mammary tumour in paraffin block (which includes the typing characteristics and having the largest representation of vital tumour epithelial component, *i.e*. without regressive changes such as extensive necrosis) was chosen for immunohistochemical analysis. The detection of selected markers of mechanism of action (mentioned below) was carried out by indirect immunohistochemical method on whole paraffin sections, utilizing commercially available rat‐specific antibodies (Santa Cruz Biotechnology, Paso Robles, CA, USA; Dako, Glostrup, Denmark; Bioss, Woburn, MA, USA; GeneTex, Irvine, CA, USA; Abcam, Cambridge, MA, USA; Boster Biological Technology, Pleasanton, CA, USA; Thermo Fisher Scientific, Rockford, IL, USA). All steps of the immunohistochemical staining were processed according to the manufacturers’ recommendations as we described previously 13, 14. The concentration used for each primary antibody is as follows: caspase‐3 1:500, Bax 1:200, Bcl‐2 1:200, Ki‐67 1:50, VEGFA 1:150, VEGFR‐2 1:80, MDA 1:1000, CD24 1:200, CD44 1:200, ALDH1A1 1:500, H3K4m3 1:500, H3K9m3 1:400, H4K20m3 1:300, H4K16ac 1:200. The primary antibodies were visualized by a secondary staining system (EnVision, Dual Link System‐HRP, cat. no. K060911, Dako North America, Carpinteria, CA, USA) using diaminobenzidine tetrahydrochloride (DAB) as a substrate. Negative controls included omission of primary antibody. Immunohistochemically detected antigen expression was evaluated by precise morphometric method. Sections were screened, and digital images at magnifications of ×400 were microscopically analysed (Olympus BX41N). Expression of proteins was quantified as the average percentage of antigen‐positive area in standard fields (0.5655 mm^2^) of tumour hot spot areas 15. Morphometric analysis of the digital images was performed using QuickPHOTO MICRO software, version 3.0 (Promicra, Prague, Czech Republic). The values were compared between treated (CLO 0.1 and CLO 1) and non‐treated (control) tumour cells of female rats; at least 60 images for one marker were analysed (in total 840 of tumour slides for 14 markers).

### Nucleic acid extraction and bisulphite conversion

Prior to the isolation of DNA, fresh frozen tumour samples were disrupted by TissueLyser LT (Qiagen, Hilden, Germany). An average of 50–100 mg of sample and stainless steel bead 5 mm in diameter (Qiagen) were added into pre‐cooled tube. Samples were disrupted and homogenized in 200 μl of lysis buffer (Qiagen) in TissueLyser LT (Qiagen) at 50 Hz until the tissue was completely disturbed. Homogenized samples were incubated at 56°C for 4 days with addition of 20 μl of proteinase K. Genomic DNA was extracted using the DNeasy Blood & Tissue Kit (Qiagen) according to the manufacturer's protocol. DNA concentration was estimated by the Qubit^™^ 3.0 Fluorometer (Thermo Fisher Scientific, Waltham, MA, USA) at a wavelength of 260 nm. At least 50 ng DNA was used for sodium bisulphite modification using an EpiTect Bisulfite Kit (Qiagen) according to the manufacturer's recommendation.

### Quantitative methylation analysis (pyrosequencing)

Quantitative pyrosequencing was performed with the PyroMark PCR Kit (Qiagen). Predesigned methylation assays were used to determine the methylation status of three CpG sites in the RASSF1A and six CpG islands in the tissue inhibitor of metalloproteinase‐3 (TIMP3) promoter (PyroMark CpG assay; Qiagen). The total volume of PCR reaction was 25 μl including 20 ng of bisulphite‐treated DNA. Thermal cycling protocol included steps as initial denaturation at 95°C for 15 min., followed by 45 cycles of amplification: 94°C for 30 sec., 56°C during 30 sec. and 72°C for 30 sec. and a final extension at 72°C for 10 min. The amplification products were confirmed by electrophoresis on 1.75% agarose gel, stained with GelRed Nucleic Acid Biotium Inc. (Fremont, CA, USA) and visualized on UV transilluminator. Obtained PCR products were analysed according to the manufacturer's instructions using the PyroMark Q96 ID System (Qiagen) with PyroMark Gold Q96 Reagents. Methylation data were evaluated with the instrument software (PyroMark Q96 software, version 2.5.8; Qiagen).

### Cell culture and Experimental design

The human cancer cell line MCF‐7 (human breast adenocarcinoma, ECACC, Porton Down, Salisbury, UK) was cultured in Dulbecco's modified Eagle's medium with Glutamax‐I and sodium pyruvate (GE Healthcare, Piscataway, NJ, USA) supplemented with a 10% foetal bovine serum, penicillin (100 IU/ml) and streptomycin (100 μg/ml) (all Invitrogen, Carlsbad, CA, USA) in an atmosphere containing 5% CO_2_ in humidified air at 37°C. Cell viability, estimated by trypan blue exclusion, was greater than 95% before each experiment.

MCF‐7 cells (3 × 10^5^) were seeded in Petri dishes (60 × 15 mm) and cultivated 24 hrs in a complete medium with 10% FCS. Cells were treated with CLO ethanol extract (Calendula, Nová Ľubovňa, Slovak Republic) for 24, 48 and 72 hrs prior to analysis. CLO plant extract solution was prepared from powder (*Syzygium aromaticum*) diluted in 40% ethanol. Ethanol final concentration in experimental groups containing CLO extract (c = 350 resp. 450 μg/ml) experiments was max. 0.4% with no toxicity (data not shown). To exclude toxicity of vehicle solution (ethanol), we perform several dilutions in a range comparable to final concentrations in CLO extract. CLO extract concentration 600–1000 μg/ml (0.5–0.8% ethanol concentration) in experimental groups showed ethanol toxicity (data not shown) therefore was excluded from further experiments.

### Cytotoxicity assay

The MTS colorimetric assay 16 was used to determine cytotoxic effects of CLO extract at final concentrations of 50–1000 μg/ml. After 72 hrs of incubation, 10 μl of MTS (Promega, Madison, WI, USA) was added to each well according to the CellTiter 96^®^ AQueous One Solution Cell Proliferation Assay protocol. After minimum 1 hr of incubation, the absorbance was measured at 490 nm using the automated Cytation^™^ 3 Cell Imaging Multi‐Mode Reader (Biotek, Winooski, VT, USA). The absorbance of the untreated control wells was taken as 100%, and the results were expressed as a percentage of the control. All experiments were performed in triplicate including untreated controls and vehicle (ethanol) internal controls. For following analyses, final concentration 350 μg/ml was used.

### 5‐Bromo‐20‐deoxyuridine (BrdU) cell proliferation assay

The BrdU incorporation was used to analyse cell proliferation activity monitored by quantification of BrdU introduced to the genomic DNA during cell growth after 72‐hrs CLO extract (c = 50–1000 μg/ml) treatment. DNA synthesis was assessed using colorimetric cell proliferation ELISA assay (Roche Diagnostics GmbH, Mannheim, Germany) following manufacture protocol. The colour intensity was measured with Cytation^™^ 3 Cell Imaging Multi‐Mode Reader (Biotek) at 450 nm (reference wavelength: 690 nm). The results were expressed as a percentage of the control. All experiments were performed in triplicate including untreated controls and vehicle (ethanol) internal controls. For following analyses, final concentration 450 μg/ml was used.

### Flow cytometry analysis protocol

For flow cytometric analysis (FCM), floating and adherent cells were harvested together 24, 48 and 72 hrs after treatment (CLO final concentrations 350 and 450 μg/ml), washed in PBS, resuspended in PBS and stained prior to analysis as described previously 13, 14. Fluorescence was detected, after 15‐ to 30‐min. incubation in the dark at room temperature, using a FACSCalibur flow cytometer (Becton Dickinson, San Jose, CA, USA).

### The examinations of secondary metabolites in CLOs ethanol extract

GC‐MS analysis. GC/MS of essential oil was performed using Thermo Finnigan GC: Trace GC and MS: Trace DSQ (USA), equipped with a fused‐silica capillary column **(**25 m × 0.20 mm; 0.33 μm film thickness, model HP‐5MS, Agilent Technologies, Santa Clara, CA, USA). Column temperature was set initially at 60°C for 8 min., and then, heating was programmed from 60 to 260°C at 8°C/min up to 70°C; 5°C/min up 80°C; 4°C/min up 260°C and holding at 260°C for 1 min. The injector was maintained at 280°C, and helium was used as the carrier gas (constant flow 1 ml/min.). Ion source temperature was 200°C. The ionization energy was 70 eV with a mass range of 50–300 AMU. The volatile compounds were identified by matching their mass spectra in NIST database.

LC‐MS‐DAD analysis. The LC‐MS analyses were performed on an Agilent 1260 Infinity LC System (Agilent Technologies, Santa Clara, CA, USA), equipped with a binary pump, an autosampler, a column thermostat and a diode array detector (DAD), coupled to a quadrupole time‐of‐flight (6520 Accurate‐Mass QTOF) instrument equipped with an orthogonal ESI source (Agilent Technologies). HPLC separation of clove extract was carried out on a Kromasil C18 column (4.6 × 150 mm, 5 μm, Sigma‐Aldrich) at 35°C and a flow rate of 0.4 ml/min. Water (pH 3.1) and MeCN were used as mobile phases A and B, respectively. The method was performed according to Fialová *et al*., 2015. Phenolic compounds were identified by comparing their UV and mass spectra with literature and authentic standards when available and by measuring accurate m/z. The quantitative determination of phenolic compounds in clove's extract was provided by the method of external standards.

### Statistical analyses

In *in vivo* study, data are expressed as means ± S.E.M. The Mann–Whitney test, Kruskal–Wallis test, Student's *t*‐test and one‐way analysis of variance (anova) were the statistical methods used in data evaluation. Tumour volume was calculated according to the formula: $ \hbox{V} = \pi. (\hbox{S}_{_1_^}^)^^2^ . S_2_ / 12 (S_1_, S_2_ are tumour diameters; S_1_ < S_2_). In florescence assay, anova was first carried out to test for the differences between groups; comparisons between individual groups were made using a Student–Newman–Keuls multiple‐comparisons test. The median levels of methylation in CpG islands in CLO 0.1 and CLO 1 were compared to the median levels in controls by the Wilcoxon rank‐sum test, using the R software. In *in vitro* study, data are expressed as means ± S.D. Data were analysed using anova followed by the Bonferroni multiple‐comparisons test. Differences were considered significant when *P* < 0.05. The examinations of secondary metabolites in clove's ethanol extract were performed in triplicate. The quantitative results were calculated from calibration curves, expressed as means ± S.D. Data analyses were conducted using graphpad prism, version 5.01 (GraphPad Software, La Jolla, CA, USA).

## Results

### Parameters of mammary carcinogenesis in rats and histopathology of tumours

**Figure 1:**
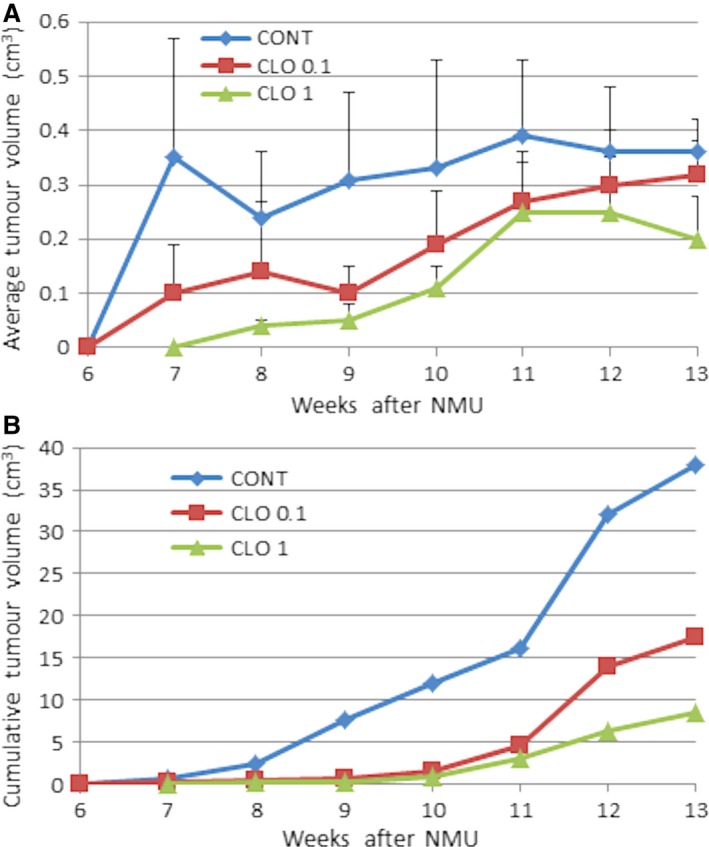
Changes in average and cumulative tumour volume during the study. Data are expressed as means ± S.E.M. (**A**); data are expressed as a sum of volumes per group (**B**).

CLO significantly inhibited the risk of mammary gland carcinomas in rats in a dose‐dependent manner (Table 1). Higher dose of dietary administered CLO significantly decreased tumour frequency by 58.5% compared to control rats. In the same experimental group, tumour latency, incidence and average tumour volume were not changed significantly in comparison with control group. The treatment efficacy (tumour frequency) observed in the CLO 1 group significantly correlated (*r* = 0.473, *P* < 0.001) with the decrease in average tumour volume. Changes in tumour volume in each week of the study is showed in Figure 1A and B. Lower CLO dose significantly decreased tumour frequency by 47.5% (*P* = 0.032) in comparison with control animals.

**Table 1:** Effects of cloves in N‐methyl‐N‐nitrosourea‐induced mammary carcinogenesis in female Sprague‐Dawley rats at the end of experiment

Group	CONT	CLO 0.1	CLO 1
Total number of mammary tumours	105	55	42
HG/LG carcinoma	12 / 87	8 / 45	6 / 34
Tumour bearing animals / all animals	21 / 25	20 / 25	21 / 24
Tumour frequency per group^a^	4.20 ± 0.80	2.20 ± 0.42* (−47.5%)	1.75 ± 0.26**(−58.5%)
Tumour latency^a^ (days)	69.33 ± 2.97	75.25 ± 2.96 (+ 6 days)	76.67 ± 3.19 (+ 7.5 days)
Tumour incidence (%)	84.0	80.0 (−5%)	87.5 (+ 4%)
Average tumour volume^a^ (cm^3^)	0.36 ± 0.07	0.32 ± 0.08 (−11%)	0.20 ± 0.08 (−44%)
Cumulative tumour volume^b^ (cm^3^)	37.95	17.37 (−54%)	8.47 (−78%)

CONT—control group, CLO 0.1—group with administered cloves at a concentration of 1 g/kg in diet, CLO 1—group with administered cloves at a concentration of 10 g/kg in diet.

^a^Data are expressed as means ± S.E.M.

^b^Data are expressed as a sum of volumes per group. Values in brackets are calculated as %‐ual deviation from the 100% of non‐influenced control group (with exception of latency) .

Significantly different, *P < 0.05, **P < 0.01 *versus* CONT.

The cribriform carcinomas, mixed cribriform/papillary and papillary/cribriform carcinomas were the most frequent mammary lesions occurred in experiment. The rates of HG/LG carcinomas were not changed by chemoprevention.

### Immunohistochemistry of rat tumours

**Figure 2:**
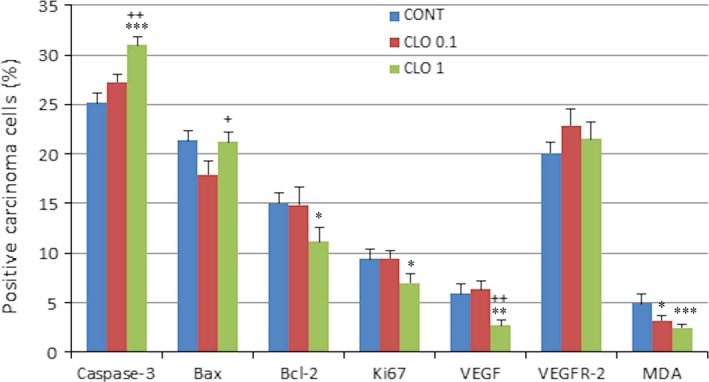
Immunohistochemical evaluation of caspase‐3 (cytoplasmic), Bax, Bcl‐2, Ki67, VEGFA, VEGFR‐2 and MDA expression in rat mammary carcinoma cells after the administration of cloves in two doses. Data are expressed as means ± S.E.M. Significantly different, *P < 0.05, **P < 0.01, ***P < 0.001 *versus* CONT, ^+^ *P* < 0.05, ^++^ *P* < 0.01 *versus* CLO 0.1. Figure represents the expression of proteins quantified as the average percentage of antigen‐positive area in standard fields (0.5655 mm^2^) of tumour hot spot areas. The values of protein expression were compared between treated (CLO 0.1, CLO 1) and non‐treated (control) carcinoma cells of rat females; at least 60 images for one marker were analysed.

Figure 2 shows the evaluation of markers of apoptosis, proliferation, angiogenesis and antioxidation effect in rat mammary carcinoma cells. In the CLO 1 group, increases in cytoplasmic caspase‐3 expression by 23% (*P* = 0.0006) and also nucleic caspase‐3 expression by 69.5% (*P* = 0.0008) were observed in comparison with the control. Higher dose of CLO significantly reduced expressions of Bcl‐2 by 26% (*P* = 0.020), Ki67 by 25% (*P* = 0.049), VEGFA by 55% (*P* = 0.0015) and MDA by 50.5% (*P* = 0.0004) compared to controls. Lower CLO dose significantly reduced tumour MDA levels by 35.5% (*P* = 0.021) when compared to controls. Bax and VEGFR‐2 expressions were not changed in treated carcinoma cells in comparison with the control.

**Figure 3:**
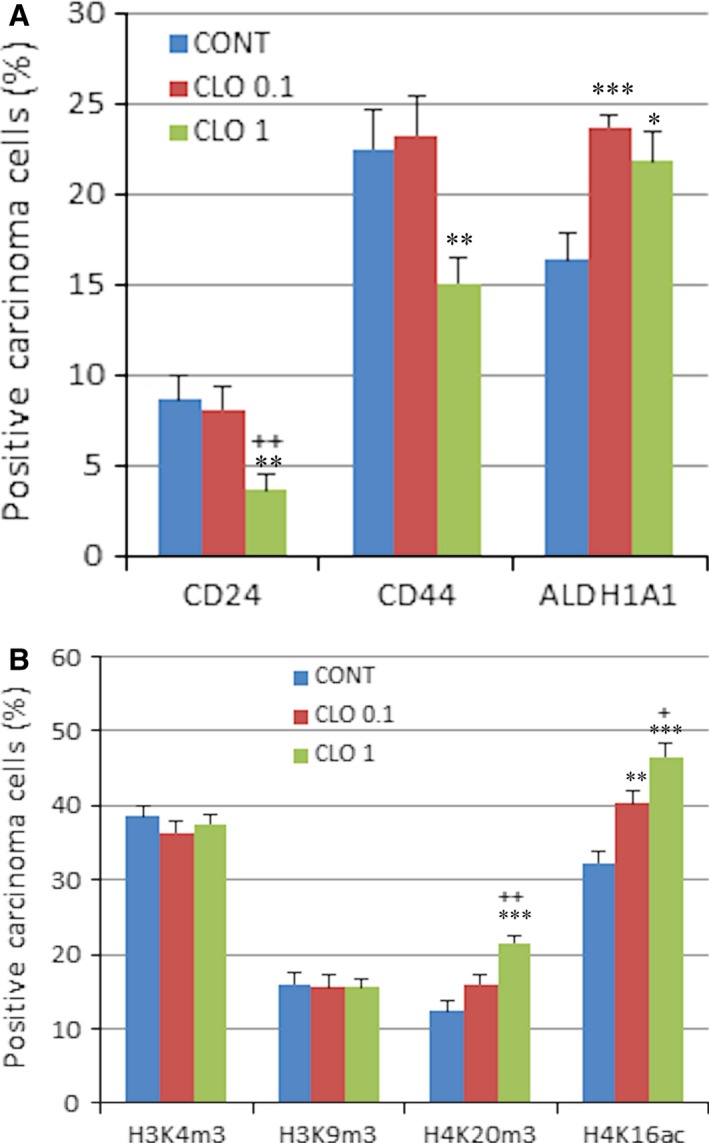
Immunoexpression of cancer stem‐cell (**A**) and epigenome (**B**) markers in rat breast carcinoma cells after the treatment with cloves. Data are expressed as means ± S.E.M. Significantly different, *P < 0.05, **P < 0.01, ***P < 0.001 *versus* CONT, ^+^ *P* < 0.05, ^++^ *P* < 0.01 *versus* CLO 0.1. The values of protein expression were compared between treated (CLO 0.1, CLO 1) and non‐treated (control) carcinoma cells of rat females; at least 60 images for one marker were analysed.

The evaluation of cancer stem‐cell parameters demonstrated significant dose‐independent increase in the ALDH1A1 expression by 44.5% (*P* < 0.0001) in CLO 0.1 and 33% (*P* = 0.012) in CLO 1 and significant decreases in CD24 and CD44 expressions (however only in high‐dose CLO) by 57.5% (*P* = 0.0035) and 33% (*P* = 0.0063) in comparison with the control carcinomas (Fig. 3A).

Results of histone 4 chemical modifications showed significant dose‐dependent increase in H4K16ac levels by 25% (*P* = 0.001) in CLO 0.1 and 44.5% (*P* < 0.0001) in CLO 1 and significant increase in H4K20me3 by 74% (*P* < 0.0001) in high‐dose CLO group when compared to controls (Fig. 3B). Changes in H3K4 m3 and H3K9 m3 levels were not significant compared to control group.

**Figure 4:**
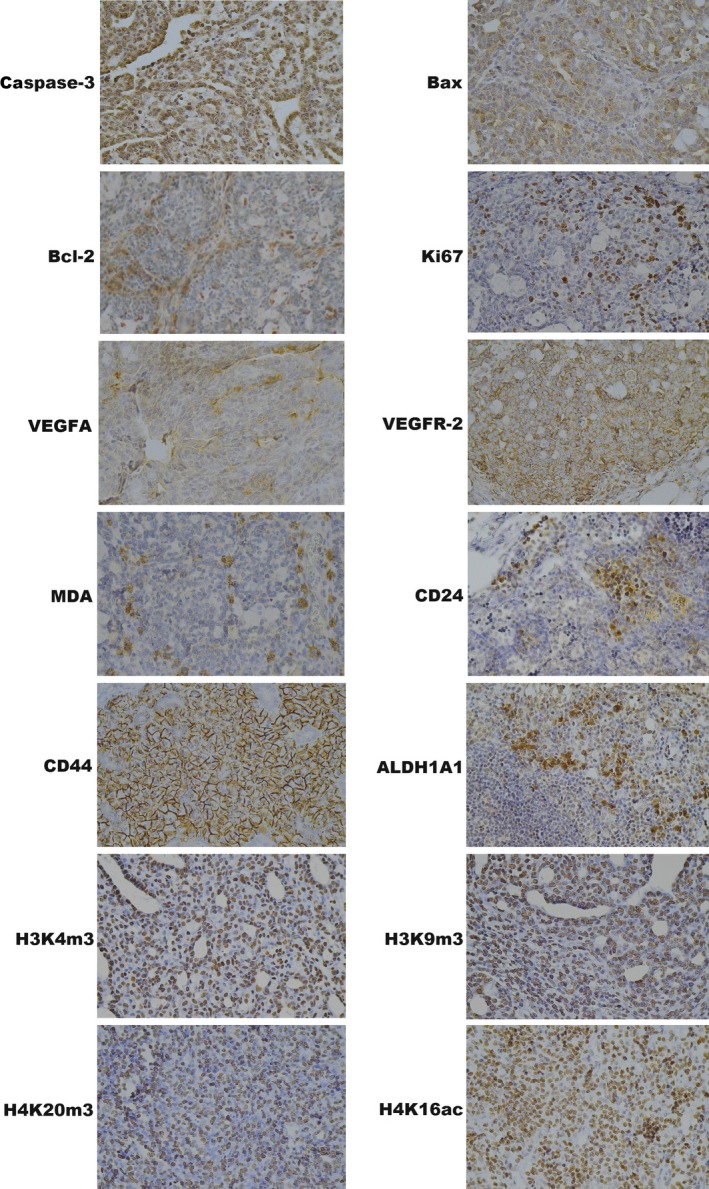
Representative images of caspase‐3, Bax, Bcl‐2, Ki67, VEGFA, VEGFR‐2, MDA, CD24, CD44, ALDH1A1, H3K4 m3, H3K9 m3, H4K20 m3, H4K16ac expressions in rat mammary carcinoma cells. For detection, polyclonal caspase‐3 antibody (Bioss, Woburn, USA), polyclonal Bax and Bcl‐2 antibodies (Santa Cruz Biotechnology), monoclonal Ki67 antibody (Dako), monoclonal VEGFA and VEGFR‐2 antibodies (Santa Cruz Biotechnology), polyclonal CD24 antibody (GeneTex), polyclonal CD44 antibody (Boster), polyclonal ALDH1A1 antibody (Thermo Fisher), polyclonal EpCAM antibody (Abcam) polyclonal H3K4 m, H3K9 m3 and H4K20 m3 antibodies (Abcam) and monoclonal H4K16ac antibody (Abcam) were used; final magnifications: ×400.

Representative pictures of the expressions of caspase‐3, Bax, Bcl‐2, Ki67, VEGFA, VEGFR‐2, MDA, CD24, CD44, ALDH1A1, H3K4 m3, H3K9 m3, H4K20 m3 and H4K16ac in mammary carcinomas of the rats are outlined in Figure 4.

### Quantitative methylation analysis

**Figure 5:**
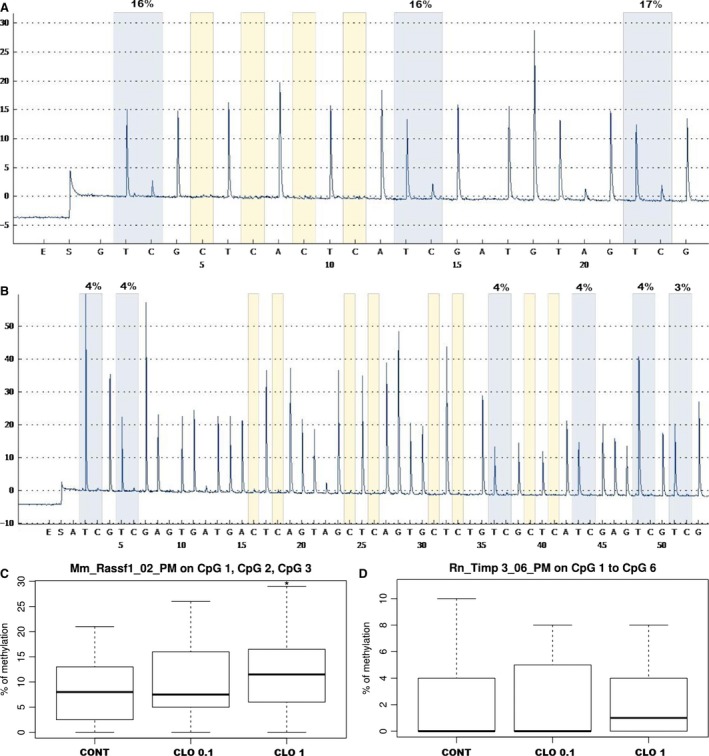
DNA promoter methylation of RASSF1A and TIMP3 genes in mammary carcinomas. Representative pyrograms of RASSF1A (**A**) and TIMP3 (**B**) promoter region (methylation in monitored CpG areas). Total promoter methylation of RASSF1A (**C**) and TIMP3 (**D**) in mammary carcinomas in control and treated groups. Significantly different, *P < 0.05 *versus* controls.

A total of 24 mammary tumour samples (divided into three groups, mentioned above) were initially included in the study to quantify the level of methylation status of three and six CpG sites in the RASSF1A (Fig. 5A) or TIMP3 (Fig. 5B) promoter, respectively.

In the case of RASSF1A methylation of individual CpG sites, there were no significant differences between the treated groups and control. Analysis of total methylation of RASSF1A promoter (including three evaluated CpG sites) showed a statistically significant increase in CLO 1 group compared to controls (*P* = 0.033) (Fig. 5C). In TIMP3 promoter, the average methylation levels of CpG3 and CpG4 islands in CLO 1 group were significantly lower (*P* = 0.016, *P* = 0.048); on the other hand, the methylation level of CpG5 site was significantly increased compared to the control group (*P* = 0.012). Comparison between controls and treated groups did not show significant differences in total methylation of TIMP3 promoter (including six evaluated sites of CpG1‐CpG6) (Fig. 5D).

### Other effects in animals

CLO dose‐dependently lowered serum levels of triacylglycerols by 16.5% (*P* < 0.05) and 19% (*P* < 0.01) in comparison with control rats. Moreover, CLO significantly lowered serum very low‐density lipoprotein (VLDL)‐cholesterol levels by 16% (*P* < 0.05) (CLO 0.1 / CLO 1 *versus* controls). In the serum, slight (but significant) increase in glucose levels by 12% (*P* < 0.01) in CLO 1 group was found (data not shown). The differences in final bodyweight were not seen among experimental groups. Treated animals showed a non‐significant increase in food intake by 1.5 g (CLO 0.1) and 2 g (CLO 1) in comparison with the control animals. Dietary administered clove buds were well tolerated by rats, and there were no macroscopic changes in the observed organs (liver steatosis, hepato/splenomegaly, gastritis) or haematopoietic disorders in the treated animals.

### *In vitro* study

**Figure 6:**
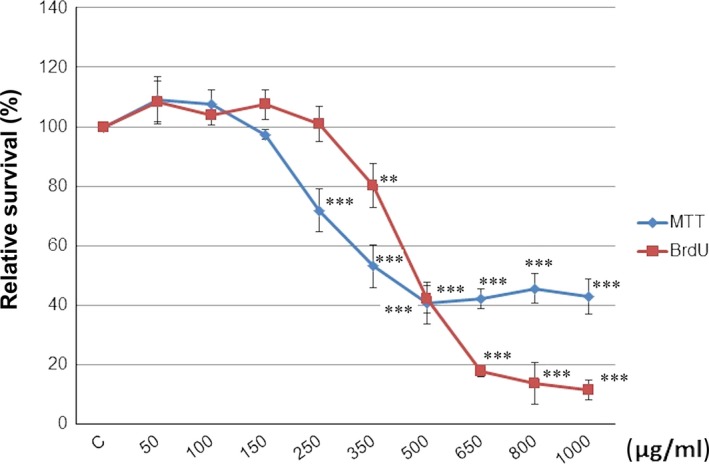
Relative survival of MCF‐7 cells treated with CLO extract (50–1000 μg/ml) and analysed by MTS and BrdU incorporation assays. Data were obtained from three independent experiments, and significant differences were marked as **P < 0.01, ***P < 0.001 *versus* control cells (untreated).

The MTS and BrdU incorporation assays were used to evaluate the CLO extract antiproliferative effect on MCF‐7 cells. Results showed that CLO extract significantly lowered the metabolic activity which was linked with decreased cell survival by a dose‐ and time‐dependent manner (Fig. 6). Depending on both analyses and IC 50 evaluation, we used two different concentrations (CLO 350 and CLO 450 μg/ml) of CLO extract to followed experiments.

Several flow cytometric analyses of MCF‐7 cells after CLO extract treatment were managed after 24, 48 and 72 hrs. Evaluation of cell cycle progression (Table 2) after CLO treatment showed significant accumulation of cells in S phase shortly after 24 hrs. After 48 hrs, unchanged S phase accumulation persists only after CLO 350 treatment. On the other hand, significant occurrence of cells in sub‐G0/G1 fraction was observed after 48 and 72 hrs of CLO 450 treatment or 72 hrs after CLO 350 treatment, respectively.

**Table 2:** The cell cycle distribution in MCF7 cells after CLO treatment

Time (h)	24			48			72		
Treatment	CONT	CLO 350	CLO 450	CONT	CLO 350	CLO 450	CONT	CLO 350	CLO 450
Sub‐G_1_	0.43 ± 0.07	3.50 ± 1.27	6.29 ± 1.08	0.71 ± 0.27	3.10 ± 1.31	12.37 ± 2.01*	0.66 ± 0.02	5.41 ± 1.77*	23.62 ± 3.44**
G_0_/G_1_	74.02 ± 0.28	55.19 ± 1.38*	49.40 ± 1.77**	68.96 ± 0.45	53.59 ± 0.20*	53.17 ± 1.84*	69.00 ± 1.34	58.74 ± 1.22*	46.86 ± 2.34**
S	11.80 ± 0.35	27.74 ± 1.96**	30.14 ± 2.18**	13.98 ± 0.23	29.19 ± 0.14**	20.85 ± 1.10*	15.02 ± 0.41	23.67 ± 0.44*	19.09 ± 2.30
G_2_/M	13.74 ± 0.56	13.57 ± 1.38	14.16 ± 1.92	16.36 ± 0.74	14.12 ± 1.33	13.61 ± 1.48	15.33 ± 0.91	12.18 ± 1.60	10.43 ± 1.88

The cell cycle distribution in MCF7 cells after CLO treatment (c = 350 and 450 μg/ml) was assessed by flow cytometry. Data are expressed as means ± S.D. of three independent experiments. The significant differences between control and CLO‐treated cells were signed as *P* < 0.05 (*), *P* < 0.01 (**), *P* < 0.001 (***).

**Figure 7:**
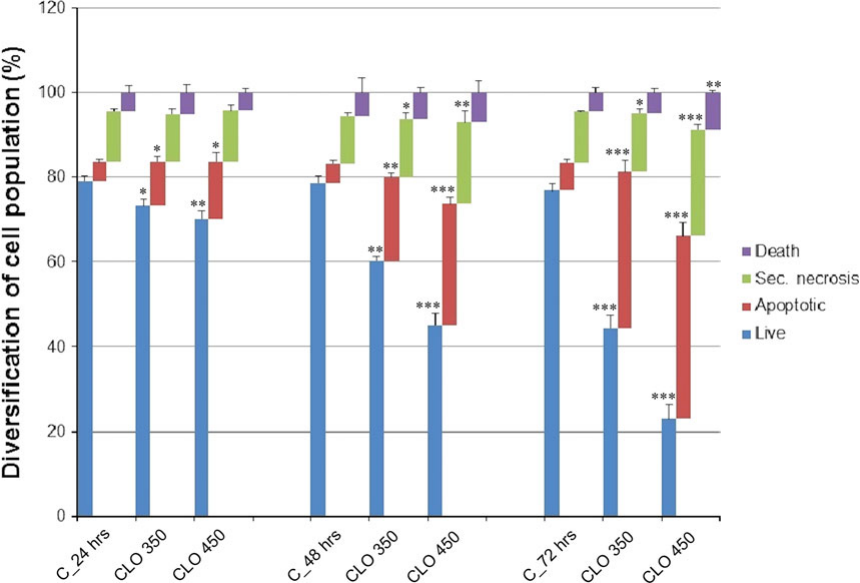
Induction of apoptosis in MCF7 cells after CLO treatment (c = 350 and 450 μg/ml) was analysed after annexin V and PI staining protocol for flow cytometry. The percentage of events in the non‐apoptotic (lower left, An−/PI−), early apoptotic (lower right, An+/PI−), late apoptotic (upper right, An+/PI+) and necrotic (upper left, An−/PI+) quadrants is indicated. Values are the means ± S.D. of three independent experiments. The significant differences between control and CLO‐treated cells were signed as *P* < 0.05 (*), *P* < 0.01 (**), *P* < 0.001 (***).

**Figure 8:**
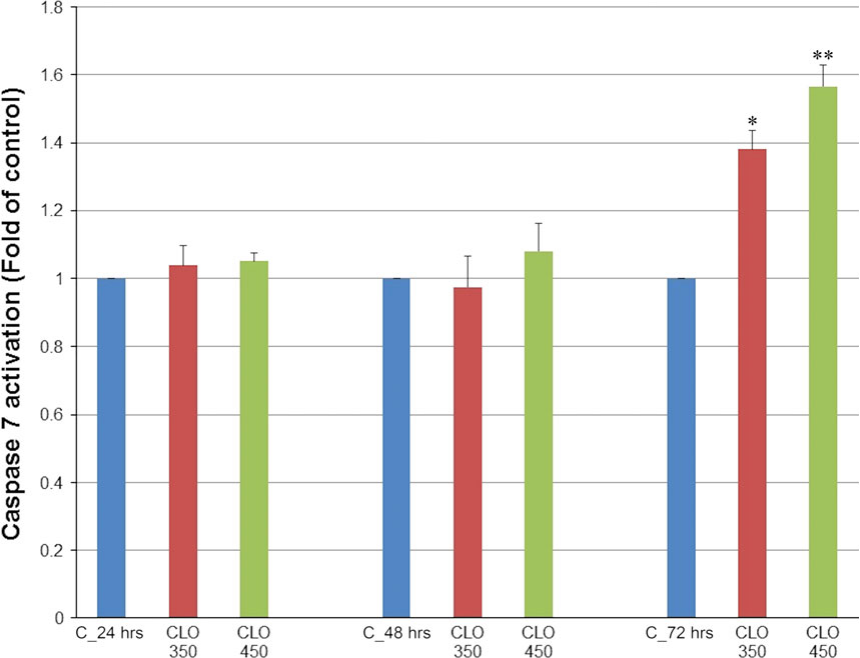
Effect of CLO extract treatment on caspase‐7 activation in MCF‐7 cell line analysed by flow cytometry. Data were obtained from three independent experiments, and significant differences were marked as *P < 0.05, **P < 0.01 *versus* control cells (untreated).

**Figure 9:**
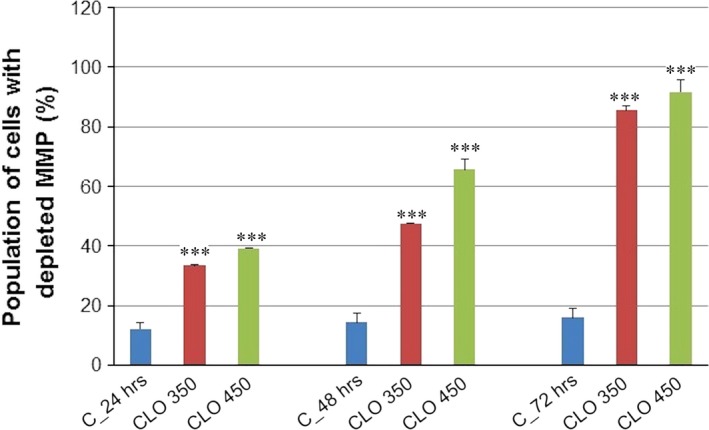
Effect of CLO extract treatment on mitochondrial membrane potential (MMP) changes. Data were obtained from three independent experiments, and significant differences were marked as ***P < 0.001 *versus* control cells (untreated).

**Figure 10:**
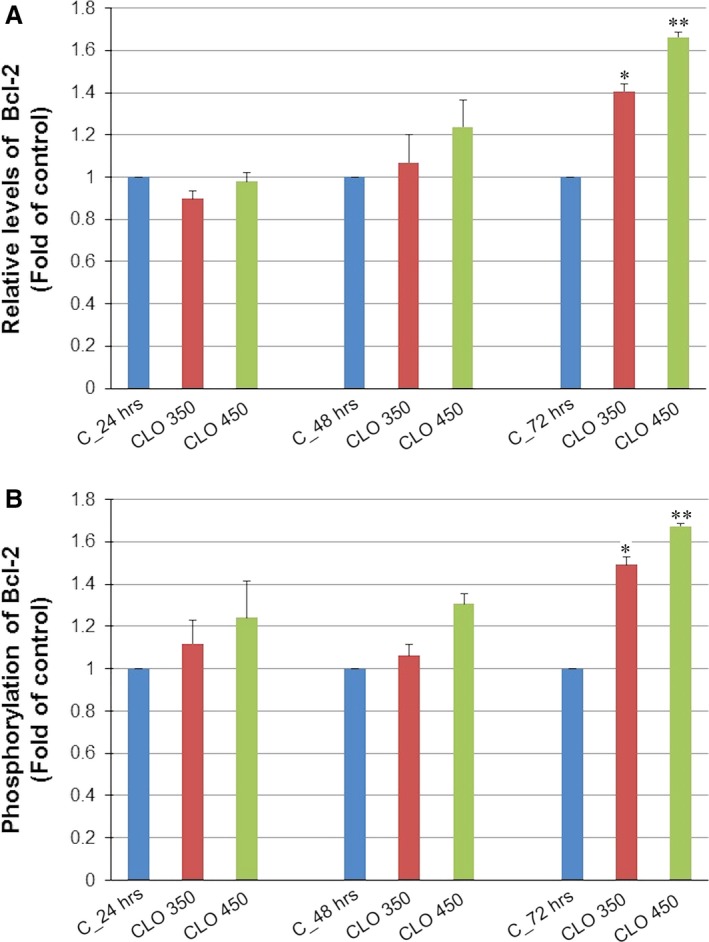
Distribution and activity of anti‐apoptotic mitochondria‐associated protein Bcl‐2 after CLO extract treatment analysed by flow cytometry. (**A**) Distribution of anti‐apoptotic protein Bcl‐2 after CLO extract treatment in MCF‐7 cells. (**B**) Deactivation of anti‐apoptotic protein Bcl‐2 by phosphorylation. Results are expressed as mean values of three independent experiments, and significant differences were marked as *P < 0.05, **P < 0.01 *versus* untreated control.

Analysis of annexin V positivity, as a marker of programmed death induction, showed significant phosphatidyl serine externalization after 24, 48 and 72 hrs of CLO 450 treatment compared with CLO 350 treatment that have delayed effect (Fig. 7). Caspase‐dependent form of cell death in MCF‐7 cells was confirmed after analysis of caspase‐7 activation (Fig. 8) after 72 hrs. Moreover, time‐dependent depletion of mitochondrial membrane potential (MMP) in MCF‐7 cells occurred after CLO extract treatment (Fig. 9). Due to mitochondrial stress after CLO extract treatment, release of anti‐apoptotic Bcl‐2 protein complexes from mitochondria to cytosol occurred (Fig. 10A). Evaluation of the phosphorylation status apparently pointed to the deactivation of Bcl‐2 anti‐apoptotic activity (Fig. 10B) and showed the activation of mitochondrial apoptosis pathway.

### Secondary metabolites in CLOs

**Figure 11:**
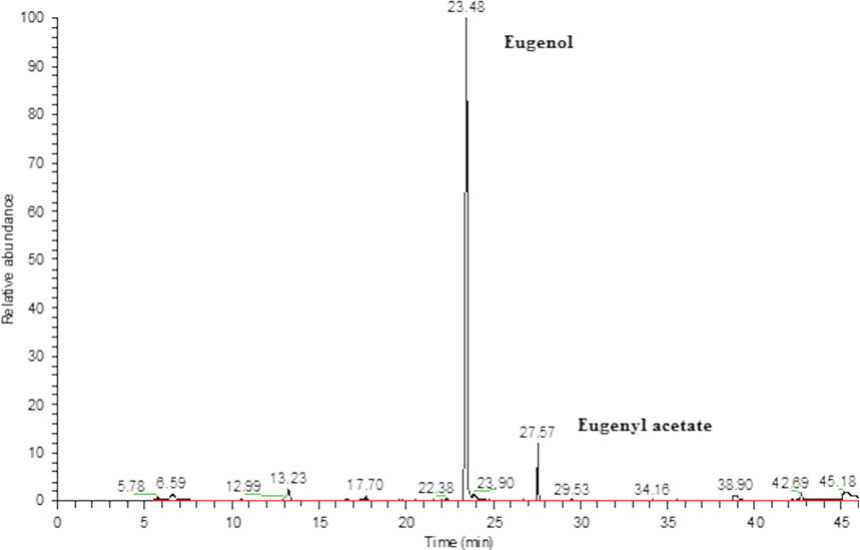
GS/MS chromatogram of clove alcohol extract with most abundant peaks.

Using LC‐MS, we identified 11 polar compounds in clove's ethanol extract. The extract consists of eugenol, organic acids and their derivatives, and flavonoids. The most plentiful metabolites were eugenol (235 mg/ml) and the derivatives of caffeoylquinic acid: chlorogenic acid (61 mg/ml) and monocaffeoylquinic acid (64 mg/ml). The qualitative and quantitative analysis of clove's ethanol extract is shown in Table 3. GC/MS analysis of clove's ethanol extract revealed the presence of two most abundant volatile compounds typical for *S. aromaticum* essential oil (Fig. 11). These compounds were identified as eugenol with relative percentage content of 80.9% and eugenyl acetate with relative percentage content of 5.4%. The rest of volatile compounds were in relative contents less than 1%.

**Table 3:** Polar phenolic compounds in clove ethanol extract, their corresponding retention times (T_R_), molecular ions [M‐H] and MS^2^ fragments in LC‐MS analysis and quantitative abundance of polar phenolic compounds (mg/ml)

	Compound	T_R_ (min)	[M‐H]^‐^ (*m/z*)	MS^2^ (20 eV) (*m/z*)	Mass concentration (mg/ml)* ± S.D.
1.	Gallic acid^a^	6.15	169	125	10.3 ± 0.05
2.	Digalloyl hexoside^a^	6.81	483	313 169	6.4 ± 0.02
3.	Galloyl‐acetylglucose^a^	16.82	373	313 169 125	3.0 ± 0.02
4.	Chlorogenic acid^b^	18.74	353	191 179	60.9 ± 0.04
5.	Monocaffeoylquinic acid^b^	23.85	353	191 179 173 135	64.3 ± 0.56
6.	Epicatechin gallate^a^	30.13	441	289 169	9.2 ± 0.02
7.	Ethylgallate^a^	35.81	197	169 125	4.6 ± 0.02
8.	Quercetin glucuronide^c^	39.28	477	301	12.3 ± 0.63
9.	Isorhamnetin hexoside^c^	51.15	463	301	7.9 ± 0.11
10.	Dimethoxyluteolin^c^	56.21	313	298	<LOQ
11.	Eugenol^d^	57.51	163	148	235.5 ± 0.71

LOQ, limit of quantification.

*Values (mg/ml liquid extract) are presented as means ± S.D. (*n* = 3), external standards: gallic acid^a^, chlorogenic acid^b^, quercetin^c^, caffeic acid^d^.

## Discussion

Strictly defined animal studies provide a high‐validity model of anticancer evaluation for novel synthetic or natural substances. The importance of plant functional foods has been well recognized in connection with health promotion and disease risk reduction, including cancer 17. Importantly, secondary metabolites of most plants as a low‐dose constituents are relatively non‐toxic with acceptable safety profile in human organism. In this study, we have focused to analyse anticancer effects of clove buds in the breast carcinoma model.

Our analysis of clove's ethanol extract indicated the presence of 11 polar phenolic compounds. This result is similar to previously published papers 9. The most abundant phenolic compound in clove ethanol extract was eugenol. The molecule of eugenol appears to possess multiple antioxidant activities (dimerization, recycling and chelating effect), thus having the potential to alleviate and prevent plethora of chronic diseases, including cancer 18. The mixture of phenolic compounds present in CLOs was markedly effective against NMU‐induced rat mammary carcinogenesis in our study. Dietary administered CLO at concentration of 0.1% and 1% in the diet significantly lowered the frequency of rat mammary carcinomas (the most sensitive parameter of rat mammary carcinogenesis) in a dose‐dependent manner by 47.5% and 58.5% compared to control animals. We suppose that the additive and synergistic activities of various phenolics present in CLOs were responsible for distinct tumour‐suppressive effects against rat mammary carcinogenesis in this study. Despite the fact that single phytochemicals demonstrated significant antioxidant and anticancer effects in several studies using different cancer cell lines 19, 20, 21, we have found that resveratrol administered alone did not indicate any anticancer effects in two repeated studies (with different doses and a way of administration) using the same model of mammary carcinogenesis in rats 22, 23. It is questionable whether an isolated plant secondary metabolite has the similar anticancer activities as the whole‐plant‐based food containing the cocktail of phytochemicals 24. In this regard, our earlier animal studies (with the same model and similar dosages as in this study) clearly demonstrated significant anticancer effects of plant foods (chlorella, fruit peels, oregano) containing mixtures of different phytochemicals manifesting comprehensive mechanism of action in the model of NMU‐induced mammary carcinogenesis in rats 13, 14, 25. Chemopreventive effects of plant foods, concretely rosemary, soya, blueberry and black raspberry in rat mammary carcinogenesis were found also by other authors 26, 27, 28, 29.

The dosing of whole‐plant‐derived foods is very specific in different species of mammals; therefore, only strictly controlled clinical trials may find appropriate dosing regimens for high‐risk humans. The lower effective dosage of CLOs in this study (0.1%) was approximately eight times higher than the usual dosage in humans, for example in Mediterranean population (0.5 g). However, both used dietary administered doses were well tolerated by animals. Doses of CLOs used in this study were based on our earlier experience with NMU‐induced rat mammary carcinoma model, where various plant‐derived functional foods were used, for example *Chlorella pyrenoidosa* 13, fruit peels 14 or oregano 25.

The mechanism of action of CLOs in this study was evaluated mainly with the immunohistochemical method, which was carried out using the precise (objective) morphometric analysis. This analysis is considered as valid method often used in human pathology. We suppose that the anticancer effect of CLOs observed in our study arises from a pro‐apoptotic, antiproliferative, anti‐angiogenic or antioxidant activities of plethora plant bioactive compounds in rat mammary gland carcinoma cells. It is well documented that the activation of *caspase*‐*3*, an executioner protease, initiates the apoptosis. There are also many other regulators of programmed cell death, for example pro‐apoptotic Bax or anti‐apoptotic Bcl‐2. Bax/Bcl‐2 ratio is considered as a good marker of the running apoptosis in cancer model studies. Some phytochemicals efficiently initiate the activation of caspase‐3 30 or raise the Bax/Bcl‐2 ratio 31 in cancer cells *in vitro*. Our experiment remarkably showed the pro‐apoptotic activities of high‐dose CLOs —it was characterized by an increase in the expression of caspase‐3 and Bax/Bcl‐2 ratio in rat mammary carcinomas. We have found similar findings in our earlier experiments reporting the pro‐apoptotic effects of Chlorella pyrenoidosa, fruit peels and oregano using the same rodent model 13, 14, 25. In our parallel *in vitro* study, we have demonstrated the decrease in a viability of MCF‐7 cell lines by clove's ethanol extract. These changes were associated with the increase in population of MCF‐7 cells in sub‐G0/G1 fraction and S phase pointing to the block in progression of cell cycle and the induction of apoptosis. Moreover, we have detected early and late stages of apoptosis or necrosis using annexin V/PI staining and also caspase‐7 activation in MCF‐7 cell lines. Finally, our *in vitro* study demonstrated the Bcl‐2 anti‐apoptotic deactivation and the mitochondrial apoptosis pathway activation through depletion in MMP.

Recent preclinical studies have shown that phenolics or terpenoids have antiproliferative and anti‐angiogenic potential in carcinogenesis 32, 33, 34, 35, 36. The expression of Ki67 is rigorously associated with the cell cycle progression 37. In this study, the decrease in Ki67 expression in rat mammary carcinomas is in accordance with our earlier results testing different plant functional foods 14, 23, 38. Our *in vitro* study (MTS and BrdU assays and analysis of cell cycle) confirmed antiproliferative effects of CLOs in breast adenocarcinoma cells. Angiogenesis, a process involving a numerous signalling pathways, is often the analysed target in cancer chemopreventive studies. The VEGF‐kinase ligand/receptor signalling has a crucial role in the formation of new blood vessels 39. We have found that the expression of VEGFA, which is an important signal molecule released by cells that promotes vasculogenesis and angiogenesis, was apparently lowered by high‐dose CLOs in rat mammary carcinomas *in vivo*. VEGF and other growth factors (*e.g*. FGFb, TGF‐alpha) are clinically interesting targets for anti‐angiogenic interventions; however, future investigation on the benefits of whole‐plant foods or isolated phytochemicals is needed.

Disturbances in the normal redox state in cells lead to oxidative stress. Free radicals that damage important cell macromolecules play a substantial role in the etiopathogenesis of cancer 40. Recently, we have found that dietary administered young barley, rich in antioxidants (flavonoids), apparently lowered the levels of dityrosines in mammary cancer cells in rats 38. MDA is often used as a marker of the oxidative damage of lipids by free radicals 41. In this study, CLOs substantially decreased MDA in mammary cancer cells *in vivo*. Our results indicate antioxidant effects of CLOs in rat carcinomas and thus point out to potential geno‐protective mechanism of action in carcinogenesis. Investigations which affirmed pro‐oxidant activities of polyphenols, terpenoids and other natural antioxidants (related only to high doses) are mainly limited to studies using cancer cell lines *in vitro* 42, which do not consistently mimic the situation in *in vivo* systems including the human body.

It is supposed that CSCs, a small subset of cancer cells, are responsible for the initiation, promotion and progression of carcinogenesis. CSCs may be responsible for the resistance of chemotherapy, which leads to relapse and multidrug resistance in cancer disease 43, 44. Currently, the evaluation of CD44, CD24 and ALDH1 expressions is considered as the most precise methodology to identify CSCs in the breast cancer women 45, 46. Recently, both CD24 and CD44 have been proven as good CSCs markers in chemocarcinogen‐induced rat mammary tumorigenesis 47. Lower expression of CD24, CD44, ALDH1 and EpCAM is linked with better prognosis of breast carcinoma in patients 48. Our recent study showed decrease in CD24 and EpCAM expression in mammary cancer cells in rats after oregano treatment 25. Results of this experiment demonstrated decrease in CD24 and CD44 expressions in carcinomas, on the other hand increase in ALDH1 after treatment with CLOs. Despite the fact that the mechanism of anti‐CSCs action of phytochemicals is not sufficiently elucidated, our results showed that both CLOs and oregano significantly altered the clinically validated markers of CSCs in the breast. The precise mechanisms responsible for the anti‐CSCs effect of CLOs need further scientific explorations, including analysis of plentiful specific cell signalling pathways.

Several phytochemicals with antitumour properties have demonstrated the influence on epigenome of the neoplastic cells 49. Aberrant chemical modifications of histones play a relevant role in carcinogenesis 4. Alterations in methylation and acetylation of histone lysines such as H3K4m3, H3K9m3, H4K20m3 and H4K16ac have been frequently associated with breast carcinoma 3, 50. Loss of histone H4K20 trimethylation has been documented as a marker of poor prognosis in patients with breast carcinoma and is linked with the invasiveness of breast carcinoma cells in a HER2‐independent manner 51. Clinical analyses of Elsheikh *et al*. 52 disclosed low or absent H4K16ac in the majority of breast cancer cases in humans (78.9%), proposing that this change may denote an early sign of breast carcinoma. As far as we know, no papers have been published about the effect of dietary factors on histone chemical modifications in NMU‐induced rat mammary carcinogenesis. Our results showed significant dose‐dependent increase in H4K20 m3 and H4K16ac levels in tumour cells. Plethoras of cancer‐related genes are a subject to methylation‐dependent silencing. Tumour suppressor genes RASSF1A (Ras‐association domain family 1, isoform A) and TIMP3 are frequently down‐regulated in breast cancer 4. In this regard, we have evaluated the methylation of several CpG islands in the promoter regions of above‐mentioned tumour suppressor genes in mammary carcinomas. Evaluating cancer tissue, CLOs demonstrated significant effects on methylation levels in RASSF1A and TIMP3 promoters. Results from similar rat model also pointed to significant effect of extra virgin olive oil on H4K20 m3 levels and RASSF1A promoter methylation in tumour cells 4. Our results could be considered as one of the plethora of mechanisms by which phytochemicals from CLOs may prevent NMU‐induced mammary gland carcinogenesis in female rats; however, our results await further experimental validation (*e.g*. with wider spectrum of gene promoters analysed).

**Figure 12:**
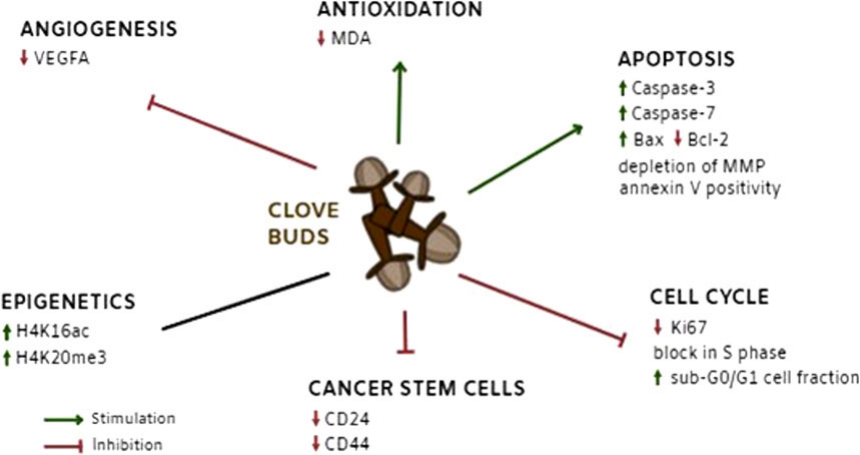
Mechanism of action of clove buds in breast carcinoma model.

Based on several different mechanisms of action of CLOs found in this study (Fig. 12), this food seems to be applicable also on other tumour types. In this regard, antiproliferative and pro‐apoptotic effects of CLOs (*Syzygium aromaticum*) in several cancer types (using different models) were observed. For instance, Dwivedi *et al*. 10 analysed the cytotoxicity effects of different *Syzygium aromaticum* extracts (MTT assay, morphological analysis and DAPI staining) using HeLa (cervical cancer), MCF‐7 (ER^+^) and MDA‐MB‐231 (ER^‐^ breast cancer), DU‐145 (prostate cancer) and TE‐13 (oesophageal cancer) cell lines. Water, ethanol and oil extracts demonstrated different patterns of cell growth inhibition activity, with the oil extract having maximal cytotoxic activity. Maximum cell death occurred in TE‐13 cells, whereas DU‐145 cells showed minimal cell demise. On the other hand, no significant cytotoxicity was found in normal human peripheral blood mononuclear cells at the same doses. Moreover, the chemopreventive potential of CLOs against benzo[a]pyrene‐induced lung carcinogenesis in strain A mice was found 53. The anticancer effects of CLOs in this study were accompanied by the up‐regulation of expression of pro‐apoptotic proteins p53, caspase‐3 and Bax, and down‐regulation of expression of anti‐apoptotic protein Bcl‐2 and growth‐promoting proteins cyclooxygenase‐2, cMyc and H‐ras.

Epidemiological studies did not sufficiently support the conception that whole‐plant (functional) foods may decrease the cancer risk so far. However, this study—for the first time—demonstrated significant anticancer activity of CLOs in mammary gland carcinoma model using both *in vivo* and *in vitro* methodological approaches. The results obtained pointed out to significant pro‐apoptotic, antiproliferative, anti‐angiogenic and antioxidant effects of CLOs in mammary carcinoma model. Moreover, this is the first study to confirm specific influence of CLOs on histone modification patterns in cancer cells *in vivo*. Based on our result, it can be concluded that regular consumption of CLOs (several times per week) could be beneficial for the risk reduction of breast cancer in humans. The determination of dosing and undesirable side effects of long‐term administration of CLOs in humans will require reliable clinical validation. However, based on the observation among Mediterranean population, daily doses of CLOs about 0.5 g consumed three to four times per week are generally considered as safe. Further research, including well‐designed and controlled clinical studies, focused on the cancer‐risk‐reducing effects of plant functional foods is warranted.

## Conflict of interest

The authors state that there are no conflicts of interest.
